# Improving Diabetes Management in Emerging Adulthood: An Intervention Development Study Using the Multiphase Optimization Strategy

**DOI:** 10.2196/20191

**Published:** 2020-10-20

**Authors:** April Idalski Carcone, Deborah A Ellis, Susan Eggly, Karen E MacDonell, Samiran Ghosh, Colleen Buggs-Saxton, Steven J Ondersma

**Affiliations:** 1 Department of Family Medicine and Public Health Sciences School of Medicine Wayne State University Detroit, MI United States; 2 Population Studies and Disparities Research Program Karmanos Cancer Institute Wayne State University Detroit, MI United States; 3 Department of Pediatrics School of Medicine Wayne State University Detroit, MI United States; 4 Division of Public Health Department of Obstetrics, Gynecology, and Reproductive Biology Michigan State University East Lansing, MI United States

**Keywords:** emerging adults, type 1 diabetes, self-determination theory, motivational interviewing

## Abstract

**Background:**

Poor diabetes self-management in emerging adulthood (age 18-25 years) is associated with poorer diabetes health and diabetes complications. Emerging adults’ focus on individuation and independence underlies their poor diabetes outcomes, offering a lever for behavior change. Self-determination theory (SDT) suggests that interventions leveraging emerging adults’ innate developmental need for autonomy may offer a route to improving diabetes outcomes by increasing feelings of responsibility for and control over diabetes self-management activities.

**Objective:**

This research project will use the multiphase optimization strategy to test the efficacy of three autonomy-supportive intervention components to elicit a clinically significant improvement in metabolic control, assessed by a 0.5% improvement in hemoglobin A_1c_ (HbA_1c_), among older adolescents and emerging adults (16-25 years) with poorly controlled type 1 diabetes (T1D; HbA_1c_≥9.0%).

**Methods:**

A question prompt list (QPL) is a tool to empower patients to assume a more active role during medical visits by asking questions and stating concerns. The motivation enhancement system (MES) is a brief counseling intervention that uses motivational interviewing communication strategies to build intrinsic motivation and self-efficacy for self-management. Text message reminders to complete diabetes care tasks may increase self-efficacy for diabetes self-management. After refining these intervention components for emerging adults, we will conduct a component selection experiment using an eight-arm full factorial design: 2 (QPL yes or no)×2 (MES yes or no)×2 (Text yes or no). Participants will complete 3 study visits: baseline, treatment end at 2 months, and a follow-up at 6 months. The primary outcome is metabolic control, which will be measured via HbA_1c_. Secondary outcomes include diabetes management and diabetes clinic attendance. SDT constructs of intrinsic motivation, self-efficacy, and the quality of the patient-provider relationship (ie, relatedness) are hypothesized mediators. Depression symptoms and emerging adults’ gender are hypothesized moderators. We will use the mixed-effects linear model for the analysis of variance of a factorial design to analyze continuous longitudinal experimental data; the generalized linear model will be used with categorical outcomes (eg, treatment attendance). The experiment was powered to detect the main effects of the intervention on the primary outcome.

**Results:**

A total of 20 participants have enrolled and completed a qualitative interview after reviewing one or more intervention components. Analysis of interview data are underway, with a report of these results anticipated in the fall of 2020. The clinical trial will be launched in the fall 2020, with participants enrolled through May 2023 and data collection continuing through November 2023.

**Conclusions:**

At the end of this experiment, we will have empirical evidence to support a large-scale, multisite effectiveness trial of an intervention package that has been optimized for older adolescents and emerging adults with poorly controlled T1D.

**Trial Registration:**

ClinicalTrials.gov NCT04066959; https://clinicaltrials.gov/ct2/show/NCT04066959

**International Registered Report Identifier (IRRID):**

DERR1-10.2196/20191

## Introduction

### Background

Diabetes management involves a regimen of daily blood glucose monitoring, insulin administration, and carbohydrate monitoring [1], a complex and demanding care routine that is primarily under the control of the patient [2]. Once considered a transient time of poor type 1 diabetes (T1D) management, the persistence of suboptimal diabetes management from adolescence into emerging adulthood (the unique developmental period between adolescence and adulthood, age 18-25 years [3]) is increasingly evident [4,5]. Studies of emerging adults suggest that rates of self-reported diabetes management are no different than those of adolescents [6]. Emerging adults complete fewer blood glucose checks per day and are more likely to miss insulin doses than older adults, a pattern of diabetes management associated with elevated hemoglobin A_1c_ (HbA_1c_) levels, the standard measure of glycemic control, and diabetes disease control [7]. Poor diabetes management in emerging adulthood has been attributed to factors such as a continuation of the decline in parental involvement in diabetes care that begins in adolescence [6] and the characteristic developmental focus of this age group on identity exploration, increasing independence, developing social networks, including increased peer and romantic relationships, new opportunities and choices, and becoming less reliant on parental support and oversight [3].

Entering adulthood with inadequate diabetes management increases the risk for gaps in health care [8] and overreliance on the emergency department for primary health care needs [9,10]. Consequently, the HbA_1c_ levels of emerging adults are similar to those of adolescents, with mean levels in the range of 8.4%-9.3% (SD 1.2-2.4) [6,11] and an estimated 83% of emerging adults failing to meet glycemic control recommendations [11]. Further, poor metabolic control is not the only consequence of inadequate diabetes management [12,13]; it is also associated with short- and long-term diabetes complications, which can appear as early as 5 years post diagnosis [14]. Thus, emerging adulthood and the period immediately preceding it are critical times for intervention. Despite this, no intervention study specifically targeting older adolescents’ and emerging adults’ T1D self-management has demonstrated improvement in diabetes management or health outcomes [15].

The developmental need for autonomy is particularly salient during late adolescence and early adulthood [3,5], making this an optimal time for interventions focused on improving capacity for independent self-management. The proposed study will test a new intervention designed to align with emerging adults’ developmental need for autonomy based on *self-determination theory* (SDT), an empirically derived theory of human motivation. SDT posits that autonomous (ie, self-initiated, driven by intrinsic vs extrinsic motivation [16]) behavior depends upon the fulfillment of 3 innate psychological needs: autonomy, or the perception that one’s behavior is self-directed; competence, or self-efficacy; and relatedness, or the existence of caring relationships supportive of the behavior [17,18]. Interventions grounded in SDT have been empirically linked to enhanced feelings of autonomy [19-21] and competence [21] as well as improvements in glycemic control [20-22] and related health outcomes [19] among adults with diabetes. Among adolescents and emerging adults with diabetes, interventions to improve self-management and glycemic control have been few. Husted et al [23] found that guided self-determination delivered by diabetes clinicians in a clinic setting increased adolescents’ perceptions of autonomy and decreased amotivation for diabetes self-management but did not improve metabolic control. Autonomy-supportive T1D camps increased adolescents’ sense of relatedness but did not change autonomy and competence; glycemic control was not examined [24]. Neither study examined the effect of autonomy-supportive interventions on diabetes self-management behavior. Most interventions targeting older adolescents and emerging adults have focused on strengthening family and peer support for diabetes management or on addressing psychological barriers (eg, mood) [25]. Few have targeted older adolescents’ and emerging adults’ own sense of responsibility for and control of their own health.

### Aims

**Figure 1:**
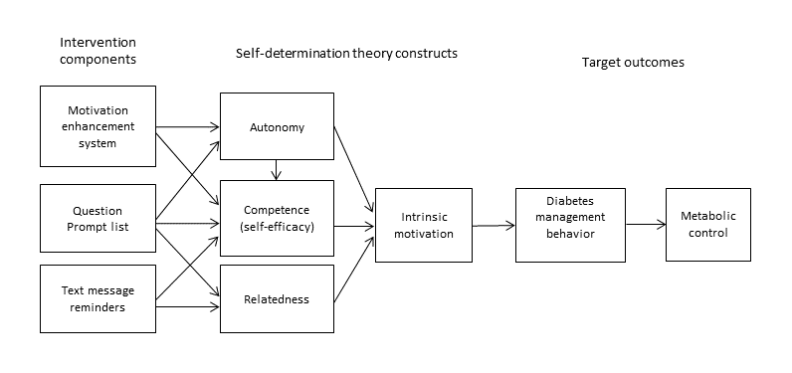
Theoretical model of the proposed intervention components.

In this paper, we present the protocol for a research project (NIH R01DK116901; Multimedia Appendix 1). The goal of this project is to develop an optimized, guided eHealth autonomy-supportive intervention to improve metabolic control through improved diabetes self-management among older adolescents and emerging adults (16-25 years) with poorly controlled (HbA_1c_≥9.0%) T1D. We have developed three self-management intervention components with theoretical and empirical links to SDT, each of which can function independently or in combination with the other components. The first is a question prompt list (QPL), a simple, inexpensive communication tool comprising a list of questions related to the physical and psychosocial aspects of illness and treatment that patients may want to ask their physicians during a clinic visit [26,27]. Theoretically, participating actively during medical visits increases patients’ feelings of control (autonomy) and competency (self-efficacy), which, in turn, empowers them to actively complete their medical care outside of medical visits. Adult cancer patients who arrive at their medical visit prepared with a QPL ask more questions and state more concerns assuming a more active role during medical visits [28-30]. There are no published studies using the QPL with older adolescents and emerging adults with T1D; however, a study of adolescents with asthma found that QPL increased confidence and helped adolescents think of and remember to ask their provider questions [31]. The second component is a brief counseling intervention, the motivation enhancement system (MES). MES uses communication strategies derived from motivational interviewing (MI) [32-34] to directly enhance intrinsic motivation and self-efficacy for self-management. Emerging adults living with HIV and asthma reported that the MES intervention increased their motivation to engage in health behaviors [35,36], adherence to medical regimens [37,38], and associated health outcomes [37,39]. MES improved parental monitoring of preadolescents’ T1D care and glycemic control [40]. Preadolescents reported improved motivation (importance) for diabetes care, greater empowerment to complete diabetes care, and enhanced support from family [41]. The third component is automated text message reminders to complete diabetes care. Engaging patients between routine diabetes clinic visits via text may increase self-efficacy for diabetes self-management [42-46]. Doing so also generates feelings of social support [47] and a caring relationship (relatedness) even when patients know the text messages are automated [44]. Figure 1 illustrates the theoretical model guiding the intervention.

The primary aim of this study is to test the efficacy of the QPL, MES, and text intervention components to improve older adolescents’ and emerging adults’ metabolic control (primary outcome) and diabetes management behavior (secondary outcome). We hypothesize that at the end of treatment (2 months) and at follow-up (6 months), older adolescents and emerging adults with poorly controlled T1D who receive one or more of the intervention components will demonstrate a clinically significant improvement in metabolic control (improvement in HbA_1c_≥0.5%) and a statistically significant improvement in self-reported and objectively measured (frequency of blood glucose monitoring) diabetes management behavior. Secondary aims include examining whether changes in SDT constructs (self-reported autonomy, self-efficacy, and patient-provider relationship) mediate intervention effects on primary outcomes at the end of treatment (2 months) and at follow-up (6 months). We also aim to explore whether treatment participation improves diabetes clinic visit attendance and whether gender and depressed mood moderate intervention effects.

## Methods

### Design

**Figure 2:**
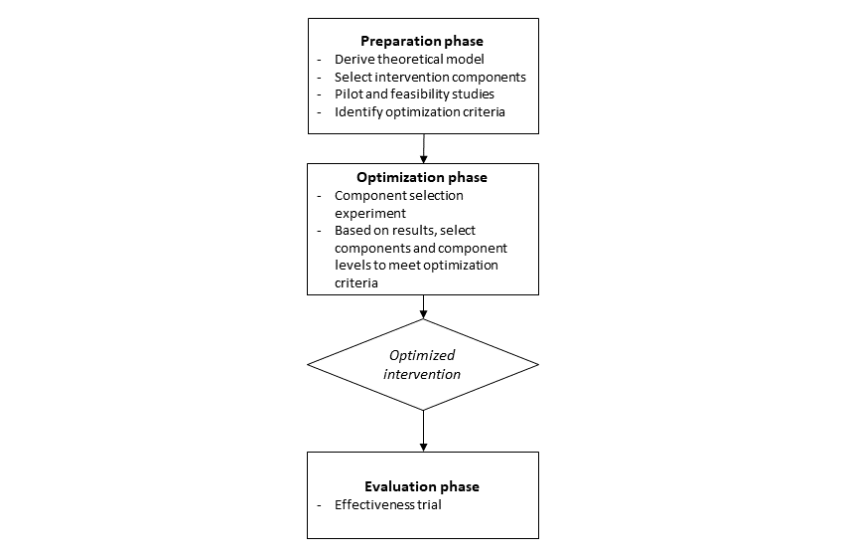
The multiphase optimization strategy study design.

This study will use a factorial trial model following the *multiphase optimization strategy* (MOST) [48,49]. The MOST design is an efficient approach to develop a multicomponent intervention in which the final intervention components are tested against an a priori defined optimization criteria. The MOST design involves 3 phases: preparation, optimization, and evaluation (Figure 2 [49]). In the *preparation phase*, a theoretical model for intervention is derived, intervention components are selected, the optimization criteria for intervention component selection are identified, and preclinical pilot and/or feasibility studies may be undertaken. In this study, we will invite members of the target population (ie, emerging adults with T1D) to review and provide feedback on three existing intervention components and then refine the components based on their feedback. In the *optimization phase,* we will conduct a component selection experiment using a randomized factorial research design to build an autonomy support intervention that has been optimized for efficacy. We will use a clinically significant improvement in metabolic control (decrease in HbA_1c_, HbA_1c_≥0.5%) as the optimization criterion for determining which intervention components should be retained in the multicomponent intervention. We chose efficacy as the optimization criterion because the eHealth intervention components, once developed, are relatively low cost (a common optimization criterion) to implement and sustain making a clinically significant reduction in HbA_1c_ the most persuasive optimization criterion for clinicians and potential payers. The MOST approach offers distinct advantages over the traditional multiple pilot randomized clinical trial approach. Including all participants in the analysis will enable an efficient, simultaneous investigation of the efficacy of each intervention component as well as synergies resulting from combinations of intervention components. Thus, this component selection experiment is analogous to conducting multiple pilot randomized clinical trials to evaluate the efficacy of each of the three intervention components and the combination of intervention components using only a fraction of the sample size and resources. At the end of this study, we will have empirical evidence supporting the efficacy of each intervention component and estimates of the efficacy of the intervention package as a whole to improve metabolic control, diabetes self-management, and diabetes clinic attendance. Empirical evidence from this study will inform the design of a large-scale, multisite effectiveness trial of the optimized intervention package.

**Figure 3:**
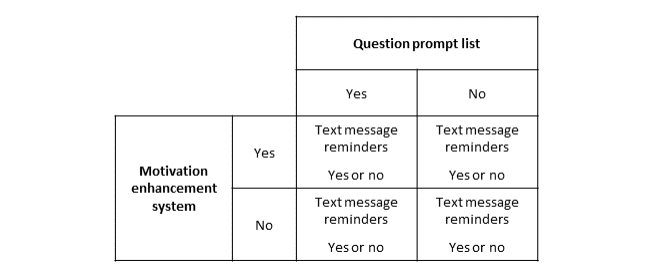
Full factorial 2×2×2 component selection experimental design in which 40 participants will be randomized to each study arm.

**Figure 4:**
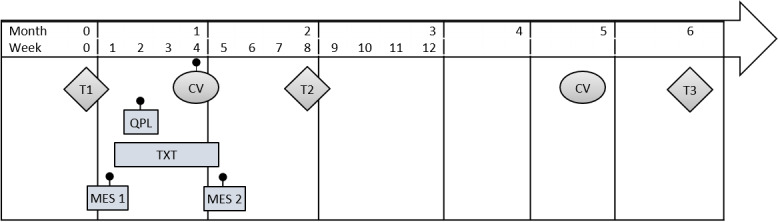
Participant timeline. CV: clinic visit; MES: motivation enhancement system; QPL: question prompt list; T: time; TXT: text message reminders.

As shown in Figure 3, the component selection experiment will use an eight-arm full factorial design: 2 (QPL yes or no)×2 (MES yes or no)×2 (text yes or no). In arms 1-3, participating youth will receive one of the three intervention components, in arms 4-6 two components; arm 7 will include all three components, and arm 8 will be the standard care control. This design will allow us to evaluate the main effect of each intervention component and explore whether combinations of components have synergy (interaction effects). The experiment, powered on the main effects, will require 320 (296 after attrition) older adolescent and young adult participants (16-25 years) with poorly controlled T1D (HbA_1c_≥9.0%). Participants will complete 3 study visits: baseline and 2 and 6 months. The intervention period is 30 days with MES session 1, and text message reminders initiated 1 week after the baseline visit. MES session 2 occurs 30 days later with the text intervention occurring between the two MES sessions. The QPL is delivered 2 weeks before the participant’s next diabetes clinic visit; hence, participants will be enrolled approximately 1 month before an upcoming diabetes clinic visit to ensure that the QPL occurs during the intervention period. The participant timeline is shown in Figure 4.

### Setting and Participants

Participants will be recruited from two Wayne State University School of Medicine sites, both located in Detroit, Michigan. We will invite eligible youth from the pediatric diabetes clinics at the Children’s Hospital of Michigan (CHM) and the adult comprehensive diabetes clinics at the Detroit Medical Center’s (DMC) University Health Center (UHC). We will target older adolescent and emerging adult patients aged 16 to 25 years, inclusive, who have been diagnosed with T1D for at least 6 months and have an elevated HbA_1c_ (HbA_1c_≥9.0% currently and averaged over the previous 6 months). We select this age range based on expert recommendations for when autonomous diabetes management is appropriate [14]. We will not exclude youth based on comorbid mental health problems (eg, depression) with the exception of conditions (ie, thought disorders, psychosis, autism, developmental delay, and suicidality) or problems of a severity that compromise data integrity, intervention participation, or youths’ ability to assume autonomous diabetes care. Nor will we exclude based on the presence of comorbid physical health problems unless the diagnosis of diabetes is secondary to another chronic medical illness (eg, cystic fibrosis) or results in atypical diabetes management. Due to the minority of non-English speaking youth at CHM and UHC, the ability to speak and read English will be required. Finally, youth will also be required to have access to a mobile device with texting capability on which they can receive the intervention components.

### Procedures

#### Recruitment and Retention

Following procedures approved by the institutional review board, we will mail a letter, cosigned by our clinician collaborators, introducing the research study to all potentially eligible youth and the caregivers of minor youth. This strategy will ensure that all eligible youth are informed of the study with adequate time to enroll. It will also permit disinterested youth the opportunity to *opt out* of being contacted regarding the study. Research assistants (RAs) will follow-up with potentially eligible youth and the caregivers of eligible minors by telephone to present the details of the study and assess their interest in participating. If the recruitment letter is returned undeliverable or RAs are unable to establish contact by phone, clinicians will introduce the study at a diabetes clinic visit and obtain a release of information and updated contact information for follow-up. RAs will obtain informed consent or, in the case of participants <18 years old, parental consent and youth assent before data collection. We will use multiple techniques to minimize follow-up attrition, including collecting detailed contact information (including three contact persons), advanced scheduling, and multiple reminder mailings and phone calls.

#### Data Collection

Given this population’s known propensity to miss clinic visits, we will conduct all study visits in youths’ homes. One month before an upcoming diabetes clinic visit, participants will have their first study visit at which the RA will obtain informed consent, baseline measurements, and download the intervention software app to the participant’s preferred device (eg, phone or tablet). The postintervention study visit will occur 2 months after baseline and is timed to occur immediately after the completion of the interventions. A second follow-up study visit will occur 6 months after baseline to assess the sustainability of intervention effects. RAs will collect self-report data using REDCap, a Health Insurance Portability and Accountability Act -compliant electronic data capture system. RAs will manually download glucose meters and extract medical chart data obtained as part of the routine medical care encounter onto paper-based forms for direct data entry. The results of HbA_1c_ tests will be similarly entered from laboratory test result forms. RAs will offer participants US $50 for completing each of the three data collections (US $150 total).

#### Randomization

Participants will be randomly assigned to one of the eight intervention conditions following their first study visit. We will stratify randomization by HbA_1c_ (high: >11.5% vs low: ≤11.5% based on the median HbA_1c_ in our prior T1D intervention studies with emerging adults). As HbA_1c_ is strongly associated with age [14], race [50], and insulin treatment [14], we effectively control for these other variables via this strategy. We will use a permuted block algorithm with blocks of eight within each HbA_1c_ stratum. Permuted blocks have the advantage of ensuring balance between treatment arms for important prognostic variables without unmasking the next treatment allocation [51]. To keep data collection staff blind to the youth’s treatment status, one RA will have exclusive data collection responsibilities. A data analyst, under our biostatistician’s supervision, will develop the randomization schedule and convey treatment assignments to the intervention coordinator who will deliver treatment assignments and initiate and monitor treatment protocols.

#### Interventions

During the first study visit, the RA will ensure that the participant can access the intervention via their preferred device (either as a mobile web app for Android devices or as a hybrid app on iOS devices) and will explain the different intervention conditions. Within 1 week of this visit, the intervention coordinator will contact youth by phone to notify them of their randomization assignment and initiate their intervention(s). Participants will be triggered to complete intervention components via text message–delivered hyperlinks. The intervention coordinator will monitor youths’ treatment completion rates, providing support and technical assistance as needed. Conversations between the intervention coordinator and youth will be audio recorded. These audio recordings will be randomly selected on a biweekly basis for the assessment of protocol fidelity. Drift from the delivery protocol will be addressed with retraining.

All three intervention components will be delivered using communication strategies derived from MI, a method of talking with people about their behavior in a way that is simultaneously client-centered and directive [34,52]. The MI framework is an autonomy-supportive intervention with strong empirical support for eliciting behavior change through intrinsic motivation. MI is consistent with SDT [53,54], as the goal of MI is to increase intrinsic motivation and self-efficacy for engaging in health-promoting behaviors [55]. In addition, emphasis on patients’ decision-making autonomy is a critical element of *MI spirit,* the relational component of MI [32-34]. The technical component of MI, that is, the use of communication techniques consistent with the MI framework [56], leads to behavior change through the elicitation of patients’ statements of intrinsic motivation (ie, *change talk*, statements about patients’ own desire, ability, reasons, and need for behavior change, and *commitment language*, patients’ statements about their intentions and plans for change). The empirical link between change talk, commitment language, and behavior change is well established [57], and evidence is growing to support the role of providers’ use of MI-consistent communication strategies in eliciting patient motivational statements [58-66]. We have previously demonstrated that clinician use of autonomy-supportive statements has been empirically linked to patient statements of intrinsic motivation [67,68]. This knowledge is integrated into the three intervention components for this study by including statements that explicitly emphasize decision-making autonomy.

##### Computerized Intervention Authoring Software

All three interventions will be developed and delivered via the Computerized Intervention Authoring Software (CIAS), version 2.0 (Interva, Inc) CIAS 2.0 is an e-intervention authoring tool that generates HTML5 mobile web apps with a responsive design capable of being deployed on any web browser and accessed via any device (eg, Apple or Android) of any size (ie, automatically reformats for optimal viewing on any size screen). As interventions built using CIAS 2.0 feature an animated narrator and a voice that reads content out loud for each screen and as iOS devices specifically disallow automatic triggering of sound files, participants with iOS devices access content via a hybrid app approach. The current mobile version of CIAS has an enhanced feature set including improved voice quality for narrated content and an updated appearance. Although mobile web and hybrid apps require internet access, 96% of Americans aged 18-29 years report consistent internet access [69]. Furthermore, technology-based interventions are ideal for youth who already have technology (cell phones and computers) integrated into their natural ecology [70-72]. Mobile web apps offer several advantages over native apps in that they do not require separate programming for different platforms, are less expensive to build and maintain, updates are centralized and automatic, they are more easily accessible and shared, and require negligible device storage space. Thus, mobile web apps exclude only a minority of youth and are consistent with trends toward ubiquitous device ownership and ready access to the internet.

##### Diabetes QPL

A QPL is a simple, inexpensive communication tool composed of questions related to the physical and psychosocial aspects of illness and treatment that patients may want to ask their physicians during a clinic visit [26,27]. QPLs are grounded in social cognitive theory, which posits that behavioral performance is largely a function of confidence in one’s ability to perform the behavior (self-efficacy) and the expectation that the behavior will result in the desired outcome [73]. Patients prepared with a QPL are more likely to ask questions and state their concerns, enabling shared decision-making and bolstering self-efficacy.

The diabetes QPL content development will be guided by the American Diabetes Association’s guidelines for diabetes treatment [14] and the empirical literature on factors that influence diabetes management during emerging adulthood. The diabetes QPL will focus on common questions about the management of T1D, various treatment options, complications, psychosocial adjustment, and transitioning to adult medical care. A diabetologist and a certified diabetes nurse educator will review the diabetes QPL for clinical relevance. Ten members of the target population will provide feedback on its relevance and acceptability via a semistructured interview. The QPL will be further refined based on this feedback.

Within 1 week of the first study visit, the intervention coordinator (an unblinded research assistant) will contact the youth randomized to the QPL by phone to explain the QPL. Approximately 2 weeks before their diabetes clinic visit, the youth will receive a text message containing a link to complete the QPL. The youth will receive reminders to complete the QPL every 3 days, escalating to daily reminders for the 3 days before the clinic visit. Upon completion, the personalized QPL will be emailed to the youth with a message reminding them to bring their QPL to their upcoming diabetes clinic visit. Additional reminders to bring the QPL to the diabetes clinic visit will be sent 1 week before and the day before the scheduled clinic visit.

##### MES

MES is a brief, computer-delivered intervention to enhance intrinsic motivation for behavior change. MES is grounded in the MI framework [32-34] and the information-motivation-behavioral skills (IMB) model of health behavior change [74]. The IMB model posits that behavior change results from the joint function of 3 critical components: accurate information about risk behaviors (eg, risks of poor diabetes self-management) or replacement health behaviors (eg, benefits of effective diabetes self-management), motivation to change behavior, and having the behavioral skills necessary to perform the behavior (eg, self-efficacy) [75]. The MES system delivers therapeutic content with high fidelity to MI principles. An animated character (avatar) guides patients through the intervention, reflecting back their responses with affirmations to boost self-efficacy and making statements emphasizing personal choice. The avatar speaks, moves/points, and displays emotional responses such as surprise, sadness, or thoughtfulness, as appropriate. The inclusion of a lifelike, synchronously interactive avatar (ethopoeia) is related to better treatment outcomes [76].

*The 3Ms* MES is a brief (>15 min), two-session mobile health intervention originally developed to improve preadolescents’ motivation for diabetes management behavior, that is, monitoring blood glucose, medication**/**insulin adherence, and meal/carbohydrate counting [41]. Session 1 begins with psychoeducation describing optimal diabetes self-management (ie, information). Youth’s motivation (operationalized as the importance of diabetes self-management) and self-efficacy (operationalized as confidence for diabetes self-management) are assessed, followed by exercises designed to increase or reinforce his/her current motivational state (eg, decisional balance) and build self-efficacy (eg, building on strengths and past success). Session 1 concludes with goal setting to promote autonomous diabetes self-management and provides the participant with optional strategies for improving diabetes management. Session 2 begins with an assessment of progress toward the behavioral goal and proceeds to build motivation and self-efficacy with exercises consistent with the youth’s current motivational state. Session 2 concludes with goal setting to promote autonomous diabetes self-management. The content of *the 3Ms* MES will be refined to be more consistent with the needs of older adolescents and emerging adults with T1D. Specifically, we will edit the avatar’s language to more strongly emphasize youths’ autonomy as it relates to diabetes self-management and edit the interactive components (ie, reasons to engage in self-management activities, potential past successes, and personal strengths/weaknesses) to be developmentally consistent with emerging adulthood. A diabetologist will review the MES for clinical relevance, and 10 members of the target population will provide feedback on its relevance and acceptability via a semistructured interview. The MES will be further refined based on this feedback.

Within 1 week of the baseline data collection, the intervention coordinator will contact youth randomized to MES by phone to explain the intervention and initiate session 1 via a link sent by a text message. Thirty days after the initial session, youth will receive a link to complete session 2. Youth will receive weekly reminders to complete the sessions until they complete the session or the intervention period has elapsed.

##### Text Message Reminders

Text message reminders (one-way) are a behavioral support strategy with theoretical support from social cognitive theory. Text message reminders promote adherence by increasing the likelihood that health-related tasks are completed, which leads to perceptions of control over health behavior and supports goal attainment [77]. We will refine a one-way text messaging protocol previously developed and evaluated with young adults with moderate to severe persistent asthma [39]. Youth will receive 30 days of one-way text message reminders. Messages will be tailored according to the youths’ preferred behavioral target derived from *the 3Ms* MES intervention, that is, youth may choose to receive text messages to monitor their blood glucose, take their insulin, count carbohydrates, or all 3 behaviors. They will be given the ability to opt out of text message reminders (none did in the asthma study [39]). Youth that do not opt out will receive daily text messages but will choose at what time(s) of day to receive their reminders. A diabetologist will review the text message reminders for clinical relevance, and 10 members of the target population will provide feedback on its relevance and acceptability via a semistructured interview. Text message reminders will be further refined based on this feedback.

Within 1 week of the baseline data collection, the intervention coordinator will contact youth randomized to text by phone to explain the intervention. The intervention coordinator will solicit a target behavior (ie, monitoring, medicine, meals, or all 3) using standardized language. The intervention coordinator will also finalize the reminder schedule and other logistics. Youth will then receive 30 days of one-way text message reminders consistent with their diabetes management goals and delivery preferences.

##### Standard Medical Care

All participants will continue to receive standard medical care at 1 of the 2 DMC clinical sites: CHM or the UHC comprehensive diabetes clinic. DMC’s clinical practices are consistent with the standards of T1D care recommended by the American Diabetes Association. Established patients with T1D visit a DMC diabetes clinic every 3-4 months for routine diabetes medical care provided by an endocrinologist and/or nurse practitioner.

### Measures

All measures have previously been used with adolescent populations; however, we will assess their psychometric performance before analysis.

Metabolic control is the primary outcome and will be measured using *HbA_1c_*. HbA_1c_ is an indirect and retrospective measure of average blood glucose levels over the previous 2-3 months. The Accubase A_1c_ test kit manufactured by DTI Laboratories will be used to measure HbA_1c_. This kit is United States Food and Drug Administration approved and uses a capillary tube blood collection method instead of venipuncture, making it suitable for home-based data collection by nonphlebotomists. DTI uses high-performance liquid chromatography to analyze the blood sample; the reagent solution contains 1 ml of ethylenediaminetetraacetic acid and 0.025 mom/l potassium cyanide, a blood preservative. A custom lot of test kits will be ordered to minimize variability across test kits. The Accubase test kit is comparable with HbA_1c_ obtained from venous whole blood (*r*^2^=0.987) [78].

Diabetes management is a secondary outcome and will be assessed via self-report and objective measures. The *diabetes management scale (DMS)* [79] is a self-report measure of daily diabetes care that assesses a broad range of management behaviors, including insulin management, dietary management, blood glucose monitoring, and symptom response. Questions ask “What percent of the time do you [eg, take all your insulin doses every day]?”, with a 0%-100% response scale. The DMS has been adapted for intensive insulin regimens with good internal consistency (α=.74 to .84) [80]. RAs will download *glucose monitors* to obtain objective data on the frequency of blood glucose monitoring. Data for participants using a blood glucose meter will be reported as the mean daily frequency of blood glucose testing during the 14 days before assessment. Continuous glucose monitoring data will be reported as the proportion of days the monitor was worn out of 14.

SDT constructs are mediators and will be assessed via self-report. The *treatment self-regulation questionnaire (TSRQ)* assesses the extent to which youth perceive their behavior as intrinsically (autonomous) or extrinsically (controlled externally) motivated [81]. The diabetes version of the TSRQ is valid and reliable (α=.80 to .86) [20]. Items (N=19, 7-point Likert) form 2 subscales, autonomous and controlled regulation, and an overall scale, the relative autonomy index [82]. Two versions of *Rollnick’s readiness ruler* [83] will be used to assess adolescents’ motivation to change their diabetes self-management routines. The *importance ruler* assesses individuals’ perceptions of the importance of changing their blood glucose testing frequency, taking prescribed doses of insulin, and adhering to dietary recommendations. The *confidence ruler* assesses an individual’s confidence (self-efficacy) in their ability to implement changes in self-management [33]. Both rulers use a 1 (not ready to change) to 10 (already trying to change) rating scale. Items are summed to obtain the total motivation for change score. Behavior-specific rulers have been widely used and are related to adolescent medication adherence [84], treatment dose [85], and treatment outcomes [86]. Cronbach α=.71 in previous research with the study population [87]. Self-efficacy for diabetes self-management will also be assessed using the *diabetes empowerment scale (DES)* [88] and the *perceived health competence scale (PHCS)* [89]. DES has 28 items assessing 3 domains of diabetes self-efficacy: managing the psychosocial aspects of diabetes, assessing dissatisfaction and readiness to change, and setting and achieving diabetes goals. DES is a widely used measure with demonstrated reliability (α=.96), validity [88], and sensitivity to change with improvements in HbA_1c_ [90]. PHCS (8 items, 5-point Likert) will be modified to assess perceptions of diabetes (vs health) competence. PHCS is reliable (α=.82 to .90) and valid (associated with health intentions and behavior) [89]. The patient-provider relationship will be assessed with the *health care climate questionnaire (HCCQ)* [91]. Participants use a 7-point Likert scale to rate 18 items in 2 domains: communicative support and practical support. The HCCQ is reliable (α=.87) and valid. The *patient activation scale (PAS)* was derived from the observation scale of the same name developed by Street et al [92]. The PAS consists of 19 items rated on a 5-point Likert scale comprising 2 scales, patient-centered communication and patient active participation.

Due to the comorbidity of depression and diabetes [93,94] and the moderating role of depression on self-efficacy in chronic illness self-management [95], symptoms of depression will be measured with the *Center for Epidemiologic Studies Depression Scale (CES-D)* [96]. The CES-D is a widely used, 20-item self-report scale that has been validated for use with adolescents [97].

The investigator-developed *family information form* will be used to collect demographic information, such as age, gender, race/ethnicity, family structure, and income level. Clinical data, including type of diabetes regimen (ie, traditional injections, intensive injections, and insulin pump), duration of diabetes, and other relevant clinical variables, will be extracted from the participants’ medical records. Diabetes clinic attendance will also be extracted for the 6-month periods before and after study initiation.

The *client evaluation of treatment (CET)*, an investigator-developed measure to assess participants’ perceptions of the usability, comprehensibility, comfort with, and usefulness of the intervention components, will be completed at the first follow-up data collection visits. Sample questions include “Do you feel this question list/computer session/text messaging program will be useful for you?” and “How easy was it for you to use the question list/computer session/text messaging program?”, with a 4-point Likert response scale.

### Data Analysis Plan

The data analysis plan is twofold. Qualitative interview data collected during the intervention refinement phase will be analyzed using thematic analysis. Quantitative experimental data will be analyzed using the mixed-effects linear model for the analysis of variance (ANOVA) of a factorial design to identify the intervention components that significantly contribute to a clinically significant improvement in HbA_1c_ (ie, a ≥0.5% decrease from baseline).

Framework matrix analysis (FMA) is an efficient, systematic approach to conducting thematic analysis [98]. An FMA analysis begins with the construction of a matrix in which the rows are based on content areas derived from the interview guide and the columns represent respondents. Two coders will first familiarize themselves with the data by reviewing the interview data. They then independently code the interviews by *charting* a summary of participant feedback into the matrix. Coders will meet after every interview to review and compare their matrices. Discrepancies will be resolved through a review of the audio and discussion, resulting in the construction of a final consensus-coded matrix. Together, the coders will identify emergent themes summarizing youths’ feedback. Data analysis will be ongoing during the data collection process. We will solicit feedback from up to 10 youth, stopping interviews if there is evidence of data saturation [99], that is, interviews are no longer generating new feedback.

Analysis of experimental data will begin with descriptive statistical analyses. The biostatistician will first characterize data heterogeneity and document the distributions of HbA_1c_, the primary outcome, and all secondary and exploratory outcomes (ie, diabetes management and clinic attendance). The data will be examined for out-of-range values, outliers, and abnormal values using graphical methods (eg, boxplots and histograms) and descriptive statistics. Unexpected findings will prompt the checking of raw data for accuracy of data entry and recording. The effect of the intervention components on the longitudinal measures of HbA_1c_ will be examined using the mixed-effects linear model for the ANOVA of a factorial design. This model will include a fixed effects indicator for each intervention component (QPL, MES, and text), time, and all interactions with time. Random intercepts will be used to account for the longitudinal nature of the data. Each model will include a random intercept and slope and fixed effects for treatment combinations (=2^3^) and time as well as the stratification variable (eg, high/low HbA_1c_). Before evaluating which components contribute to a potential reduction in HbA_1c_, models comparing the treatment with all three components and the control treatment will be examined to determine whether the complete intervention was efficacious. If this statistical test is significant, components resulting in a significant reduction in HbA_1c_ will be identified by examining the interactions between the main effects and time using the strategy advocated by Collins et al [100], which begins with the simplest effects and only adding higher-order interactions if needed. Significance thresholds will be set at α=.05 for the test of total effect (difference between the treatment with all three components and the control treatment) and α=.1 to identify which components contribute to the total effect. A higher alpha value will be used for the component selection test because it reduces the likelihood of not selecting a component that contributes to the total effect. Secondary and exploratory outcomes (diabetes management and treatment attendance) will be analyzed using a similar approach but are not powered. As treatment attendance is not a continuous outcome, a generalized linear model will be employed.

The power analyses examined the sample size required to detect clinically meaningful group differences using a mixed effect model. The proposed experiment quantifies the effects of the three experimental treatment components. Factorial trials are most often powered to detect the main effects of interventions, as adequate power to detect plausible interactions requires a greatly increased sample size [101]. As two primary hypotheses have been proposed, the Hochberg alpha adjustment will be used in hypothesis testing. The smaller of those sequential alpha levels of .025 was used in our estimates of the multiplicity-adjusted sample sizes [102]. On the basis of the simulation, the protocol proposes recruitment of 296 participants (37/condition) for a standardized medium effect size (Cohen *d*≥0.47). After adjusting for 10% attrition, our final projected sample size is 320 (40/condition), which is sufficient to preserve >80% power. The power analysis was completed in SAS (SAS Institute Inc) 9.3 software using the mixed linear model procedure. Strong preliminary support for each intervention component’s efficacy suggests that each intervention component will uniquely contribute to the overall intervention’s efficacy. Thus, the study has sufficient power to determine whether any combination of the intervention components is efficacious in improving older adolescents’ and emerging adults’ metabolic control (HbA_1c_, H1) or self-reported diabetes management behaviors (H2).

The role of sex and baseline depression status (high vs low) as moderators will be explored. These results will not be used for treatment decision-making but instead could guide the design of subsequent confirmatory trials (eg, inclusion/exclusion criteria). The focus will be on the magnitude of the effect, as recommended by Kraemer et al [103], not on significance. Fixed effects linear regression models will be used for the exploratory analyses of moderators. The dependent variable (HbA_1c_) will be expressed as a change from baseline to treatment endpoint. Independent variables include treatment and one hypothesized moderating effect per model. To demonstrate evidence of the effect of each hypothesized moderator, there must be a treatment by moderator interaction with *R^2^*≥.05. Treatment effect sizes will be estimated for each level of the moderator.

The hypothesis that SDT constructs (autonomy, self-efficacy, and the patient-provider relationship) will mediate intervention effects on primary outcomes at the end of treatment (2 months) and at follow-up (6 months) will also be assessed using fixed effects linear regression models. The dependent variable will be change in the primary outcome from baseline to months 2 and 6. Independent variables will include treatment and one hypothesized mediating effect (specified as change from baseline to months 2 and 6). Initially, the main effects will be tested with subsequent models examining the incremental contribution of the treatment by mediator interaction. Either a main effect of the mediator or treatment by mediator interaction would provide evidence of a mediator effect [103].

Attrition introduces bias and reduces power, precision, and generalizability [104]. To offset these threats and in keeping with the intention-to-treat principle, intervention termination and study termination will be distinguished, and all efforts to continue study assessments for the entire course of the study, even among those who do not continue with randomized treatment, will be undertaken [105]. The proposed mixed-effects models will incorporate all available data, even from subjects who do not complete the trial. Mixed-effects models yield valid inferences assuming ignorable attrition [106]. Two approaches will be used to examine the sensitivity of the assumption of ignorable attrition. First, we will use a pattern mixture model [107] to examine response to treatment among participants with various dropout patterns and implemented using a longitudinal strategy [108]. Second, we will ask subjects at each assessment session to rate their *intent-to-attend* the next assessment session on a Likert scale and, at baseline, to rate their intent to complete the study [109]. This variable will be used in sensitivity analyses as a baseline covariate. Estimates of the treatment effect from the models described above will be compared with models that also include the main effects of either dropout pattern or intent-to-attend.

## Results

At the writing of this report, intervention refinement activities are underway. As of July 2020, 20 participants have been enrolled and have completed a qualitative interview after reviewing one or more intervention components. The interventions are being further refined in response to this feedback. Analysis of interview data are underway, with a report of these results anticipated in the fall of 2020. The clinical trial phase is contingent on the intervention refinement activities and, thus, will be launched in the fall 2020. Participant enrollment is scheduled through May 2023, with intervention delivery wrapping up about 1 month later, in June 2023. Data collection activities will continue through November 2023, at which point study activities will focus on data analysis, dissemination, and preparing the next phase of the research, for example, developing an effectiveness trial proposal.

## Discussion

This research addresses the problem of poor diabetes management among adolescents that persists into early adulthood. We leverage the developmental needs of older adolescents/emerging adults for independence and autonomy in the construction of a multicomponent intervention that translates a basic social science theory, SDT, into three autonomy-supportive intervention components with demonstrated efficacy in similar populations and/or problems: a QPL, a MES (an eHealth intervention), and text message reminders. These intervention components will be vetted by the target population of emerging adults and then efficacy tested using the MOST, an efficient method of intervention development resulting in a potent, efficacious multicomponent intervention.

## Appendix

Multimedia Appendix 1 National Institute of Diabetes, Digestive, and Kidney Diseases peer reviews.

## Abbreviations

ANOVA: analysis of variance
CES-D: Center for Epidemiologic Studies Depression Scale
CHM: Children’s Hospital of Michigan
CIAS: Computerized Intervention Authoring Software
DES: diabetes empowerment scale
DMC: Detroit Medical Center
DMS: diabetes management scale
FMA: framework matrix analysis
HbA_1c_: hemoglobin A_1c_
HCCQ: health care climate questionnaire
HPLC: high-performance liquid chromatography
IMB: information-motivation-behavioral skills
MES: motivation enhancement system
MI: motivational interviewing
MOST: multiphase optimization strategy
PAS: patient activation scale
PHCS: perceived health competence scale
QPL: question prompt list
RA: research assistant
SDT: self-determination theory
T1D: type 1 diabetes
TSRQ: treatment self-regulation questionnaire
UHC: University Health Center
