# Supplementary material for: Improving Diabetes Management in Emerging Adulthood: An Intervention Development Study Using the Multiphase Optimization Strategy
Source: JMIR Res Protoc. 2020 Oct 20;9(10):e20191. doi: 10.2196/20191 (PMC7609201; doi:10.2196/20191)
Supplement: Multimedia Appendix 1 [file resprot_v9i10e20191_app1.pdf]

SUMMARY STATEMENT
(Privileged Communication)

PROGRAM CONTACT:
. Barbara Linder
(301) 594-0021
linderb@extra.niddk.nih.gov

Release Date: 06/20/2018
Revised Date:

Application Number: 1 R01 DK116901-01A1

Principal Investigator

CARCONE, APRIL MARIE IDALSKI

Applicant Organization: WAYNE STATE UNIVERSITY

Review Group: PRDP
Psychosocial Risk and Disease Prevention Study Section

Meeting Date: 06/11/2018
Council: OCT 2018
Requested Start: 10/01/2018

RFA/PA: PA18-330
PCC: DBL T1RX

Project Title: Improving Diabetes Health in Emerging Adulthood Through an Autonomy Supportive Intervention.
SRG Action: Impact Score:30 Percentile:14
Next Steps: Visit https://grants.nih.gov/grants/next_steps.htm
Human Subjects: 48-At time of award, restrictions will apply
Animal Subjects: 10-No live vertebrate animals involved for competing appl.
Gender: 1A-Both genders, scientifically acceptable
Minority: 1A-Minorities and non-minorities, scientifically acceptable
Children: 1A-Both Children and Adults, scientifically acceptable

Project Year	Direct Costs Requested	Estimated Total Cost
1	469,315	722,745
2	378,679	583,166
3	408,654	629,327
4	413,687	637,078
5	410,126	631,594
TOTAL	2,080,461	3,203,910

ADMINISTRATIVE BUDGET NOTE: The budget shown is the requested budget and has not been adjusted to reflect any recommendations made by reviewers. If an award is planned, the costs will be calculated by Institute grants management staff based on the recommendations outlined below in the COMMITTEE BUDGET RECOMMENDATIONS section.
NEW INVESTIGATOR

1R01DK116901-01A1 Carcone, April

COMMITTEE BUDGET RECOMMENDATIONS
EARLY STAGE INVESTIGATOR
NEW INVESTIGATOR
PROTECTION OF HUMAN SUBJECTS UNACCEPTABLE

RESUME AND SUMMARY OF DISCUSSION: This application requests support to develop and test a behavioral intervention that seeks to improve type 1 diabetes (T1D) self-management approaches and uncontrolled A1C in high risk, largely minority adolescents and young adults. This resubmitted work was very responsive to prior critiques resulting in work that the review panel opined offers high impact as it very significantly addresses the public health challenge of T1D treatment adherence as poor glycemic control is particularly prevalent among emerging adults. Reviewers again noted many strengths: the compelling scientific premise is exceptional with the unique and promising approach focusing on new research exploring aspects of autonomy and self-determination surrounding TID coupled with the focus on emerging adults for whom poor metabolic control remains problematic. Reviewers judged the productive Principal Investigator and stellar team of investigators exceptional, further strengthened by an exceptional research environment. The panel noted outstanding innovation inherent in the MOST factorial design and focus on autonomy and self-determination theory targeting adolescents. The excellent approach is strengthened by preliminary data promising feasibility and initial efficacy, good retention plan, inclusion of important mediational models, ideal study sample at high risk during this age transition period, the study outcome is anchored to clinically meaning changes in A1C, the rigorous MOST design, the home-based approach and focus on self-monitoring behaviors, outstanding qualitative component, and conceptual model well-grounded in self-determination theory. A minority opinion noted moderate concerns with interventions considered lacking sufficient detail, missed opportunity to assess fidelity, lack of verification of the delivery of sufficient dose, and potential issues with lack of long term effects beyond six months. The review panel as a whole mentioned a few minor weaknesses: insufficient discussion of provider (need to tailor whether internist or pediatrician)-patient communication and overall interaction, lack of implementation metrics such as receipt of text messages, the analysis missed the opportunity to stratify by continuous glucose monitoring status, and lack of pilot data specifically among adolescents with T1D. In sum, reviewers were enthusiastic about this well written work that offers high impact to develop new strategies to improve the health of emerging adult diabetics; the many strengths far outweigh the remaining weaknesses noted.

DESCRIPTION (provided by applicant): This project will use the multiphase optimization strategy (MOST) approach to test the efficacy of an autonomy supportive behavioral intervention to improve metabolic control among older adolescents and emerging adults (16-25) with T1D. Youth this age demonstrate chronic poor metabolic control that persists into adulthood leading to the premature emergence of short- and long-term diabetes complications. Developmentally, adolescence and emerging adulthood is a time when the need for independence and autonomy are particularly salient. This new intervention will leverage youths' desire for autonomy by designing an intervention to support diabetes self-management autonomy. This intervention is guided by self-determination theory (SDT) which suggests that autonomous (i.e., self-initiated, driven by intrinsic versus extrinsic motivation) diabetes management depends upon three conditions: 1) the perception that one's behavior is self-directed, 2) feelings of competence, or self-efficacy, and 3) the existence of caring relationships supportive of the behavior. We have identified three intervention components that target the SDT constructs. A question prompt list (QPL) is a simple, inexpensive tool comprised of a list of questions that patients might consider asking their health care provider during a clinic visit. QPLs empower patients to assume a more active role (asking questions and stating concerns) during clinic visits. The Motivation Enhancing System (MES) is an eHealth intervention to increase intrinsic motivation for health behavior change. MES content is based on the Motivational Interviewing (MI) framework and the Information-Motivation-Behavioral Skills (IMB) model of health behavior change which posits that

behavior change results from the joint function of three critical components: accurate information about risk behaviors or their replacement health behaviors, motivation to change behavior, and behavioral skills necessary to perform the behavior (self-efficacy). Text message reminders (TXT) are a strategy to encourage youth to complete their diabetes self-care that also lead to gains in self- efficacy and a stronger relationship with diabetes care providers through greater communication and satisfaction. We will test the efficacy of these intervention components toward improving metabolic control in a component selection experiment (N=320). The experiment will use a factorial research design with random assignment to determine which intervention components contribute to a clinically significant improvement (≥0.5%) in HbA1c. The result of this research will be an optimized, multi-component intervention with effect size estimates that will be used to inform a large scale, fully powered effectiveness trial. This theory-driven intervention will be scalable to a variety of chronic illness contexts and the knowledge gained from this research will inform self-determination theory and behavioral interventions targeting this population (for which there currently are none).

PUBLIC HEALTH RELEVANCE: This project will test the efficacy of a multi-component behavioral intervention to improve metabolic control among older adolescents and emerging adults (16-21) with T1D, a group with chronic poor metabolic control. This intervention is grounded in self-determination theory which states that a youth who believes their diabetes management is self-directed, competent, and supported by others is more likely to consistently complete their diabetes self-care. This theory-driven intervention will be scalable to a variety of chronic illness contexts and the knowledge gained from this research will inform self-determination theory and different interventions targeting this population (currently there are no interventions that directly target emerging adults).

CRITIQUE 1

Significance: 2
Investigator(s): 1
Innovation: 2
Approach: 2
Environment: 1

Overall Impact: This R01 resubmission comes from a highly accomplished early stage investigator with previous experiences and scientific productivity relevant to the current application. The Principal Investigator is surrounded by an accomplished and cohesive team of junior and senior investigators, and the clinical and research environment for this work is very strong. The application proposes a factorial trial (MOST) design to identify efficacious components of a behavioral intervention designed to target constructs of self-determination theory to improve glycemic control and self-management behaviors among emerging adults with T1D. The scientific premise of the application is very strong, as emerging adulthood is a high-risk development period in which patients struggle with glycemic control and self-management. Existing interventions have not previously targeted autonomy support, which is both innovative and provides a sound scientific premise, particularly for emerging adults. The scientific rigor is also very strong, as the MOST design is both efficient and rigorous in answering the questions posed. Many elements of the approach have been improved in this resubmission, including greater clarity about the intervention components, more details about access to the target population, and modifications to the pre-treatment refinement activities. Overall, there is high enthusiasm for this responsive resubmission. There are numerous strengths with only a handful of remaining, relatively minor weaknesses. The likely impact of this work is judged to be high.

1. Significance:

Strengths

- Poor glycemic control is prevalent among emerging adults with T1D, and this frequently continues into adulthood and is associated with a variety of adverse health consequences.
- There is a lack of efficacious interventions that meaningfully improve glycemic control in this developmentally at-risk group.
- A compelling scientific premise is offered to support the theoretical and empirical rationale for targeting autonomy using a SDT-based intervention for this developmental period.

Weaknesses

- None noted.

2. Investigator(s):

Strengths

- The Principal Investigator is a highly productive and promising ESI with relevant experience with the target population and methods proposed in this application.
- The team offers relevant experience and expertise related to each of the three domains of the intervention’s content, including use of QPLs, MI-based MES, and TXT reminders. The statistician co-investigator is involved in other adaptive trials involving MOST and SMART designs.
- There is a documented history of collaboration among team members.

Weaknesses

- None noted.

3. Innovation:

Strengths

- Existing interventions for T1D self-management conventionally target family or peer support and/or patient education, but previous interventions have not typically focused on promoting autonomy support and SDT constructs, which may be particularly salient for emerging adults with T1D.
- Although MOST is not a novel design, its application to testing an intervention for T1D adherence and self-management is innovative.

Weaknesses

- The individual treatment components are not particularly innovative by themselves.

4. Approach:

Strengths

- The 8-arm factorial design will be able to efficiently test each of the three intervention components as well as their combined effects. The MOST design is well-suited to identify efficacious elements of the intervention.
- The team’s access to adequate numbers of patients from the target population is now better documented in the application. Appropriate recruitment strategies are identified and are feasible given the established relationships with clinicians involved in the project.
- Inclusion/exclusion criteria are well-reasoned and provide appropriate balance between internal rigor and external generalizability.

- The revised application includes plans to examine potential theoretically-based mechanisms that may mediate treatment outcomes.
- The content, intensity, modality, etc. of the intervention components are much clearer in their presentation, which improves scientific rigor.
- Theoretical grounding in SDT is a strength, particularly since it may be well-suited for this developmental transition of emerging adulthood.

Weaknesses

- While the initial, pre-trial formative work has been streamlined to include only individual interviews rather than focus groups, it still would be better if intervention content had already been developed for this topic and population rather than needing to be refined from previous content/groups (e.g., cancer, asthma, younger children with T1D).
- Some elements of the intervention (i.e., QPL) require attendance to and interaction with a clinician, so failure to attend an appointment with their provider will limit the opportunity for participants to utilize this treatment component.

5. Environment:

Strengths

- Wayne State and the affiliated hospitals provide the necessary and relevant resources and infrastructure for conducting the proposed work.

Weaknesses

- None noted.

Study Timeline:

Strengths

- Timeline is reasonable appropriate, including 9 months at the start of the project to refine and finalize treatment materials. Recruitment will be spread across Years 2-5 of the project to ensure sufficient time to achieve target recruitment goals.

Weaknesses

- None noted.

Protections for Human Subjects:

Acceptable Risks and/or Adequate Protections

- Risks of the study are low, and appropriate safeguards are described.

Data and Safety Monitoring Plan (Applicable for Clinical Trials Only):

Acceptable

- The trial will convene a 4-person DSMB including content experts. The DSMB will meet on a quarterly basis.

Inclusion of Women, Minorities and Children:

- Sex/Gender: Distribution justified scientifically
- Race/Ethnicity: Distribution justified scientifically

- For NIH-Defined Phase III trials, Plans for valid design and analysis: Scientifically acceptable
- Inclusion/Exclusion of Children under 18: Including ages <18; justified scientifically
- The sample will include adolescents and young adults aged 16-25 years with T1D. Over 75% of participants will be non-White racial/ethnic minorities (mostly African American). Approximately 55% will be women. Given the focus on improving T1D outcomes among emerging adults, these sample characteristics are scientifically appropriate.

Vertebrate Animals:

Not Applicable (No Vertebrate Animals)

Biohazards:

Not Applicable (No Biohazards)

Resubmission:

- Overall, the investigators were very responsive to previous critiques. Many changes were made based on feedback that improved the scientific premise and rigor of the resubmission. Changes that were not made (e.g., inclusion of TXT in all treatment arms) was supported by a stated rationale.

Budget and Period of Support:

Budget Modifications Recommended (in amount/time)
Recommended budget modifications or possible overlap identified:

- One of the senior Co-Investigator's salary is over the NIH cap and needs to be adjusted in the budget.

CRITIQUE 2

Significance: 1
Investigator(s): 1
Innovation: 2
Approach: 1
Environment: 1

Overall Impact: This study is from a promising new/early stage investigator. The scientific premise is strong and fills a gap in addressing type 1 diabetes (T1D) control in the life course period of late adolescence and the transition to young adulthood. The innovation of the project is also high given the MOST design and the integration of self-determination theory which is particularly salient as a framework for emerging adults. The MOST design intervention targets are based on the investigative team’s previous work. Additional aspects related to scientific rigor were also high including recruitment and retention procedures, measurement of outcomes, and the analytic plan. Finally, the investigators were highly responsive to the previous review. The potential for this application to have a long term impact on the field is high.

1. Significance:

Strengths

- Poor diabetes self-management is a concern from adolescence through young adulthood. The transition to young adulthood is a key developmental time course for the delivery of interventions. This time course is associated with multiple life transitions representing less parental involvement, more autonomy, peer and social changes, and different provider care teams.
- Matching interventions with the developmental needs of adolescents and young adults (e.g., social relationships, identify exploration) is of high scientific significance.
- The investigators were responsive in providing more detail regarding key barriers.

Weaknesses

- None noted.

2. Investigator(s):

Strengths

- The investigative team is excellent. Dr. Carcone is a promising new and early stage investigator. She has experience in conducting behavioral interventions for children with chronic illnesses, with particular emphasis on those with type 1 diabetes. She has been a Co-Investigator on multiple NIH trials in this arena, and has specific expertise relevant to the current trial including MI-based interventions with caregivers and communication/digital interventions. She also was the MPI on an R21 with young adults.
- Dr. Carcone is supported by senior level researchers with expertise in pediatric psychology (Ellis), multilevel communication (Eggly), pediatric endocrinology (Buggs-Saxton).
- The team has experience recruiting and delivering interventions for the target population.
- Other investigators bring specific skills and expertise in technology and pediatric psychology interventions (MacDonnell), statistics (Ghosh).
- A communication and coordination plan was added.

Weaknesses

- None noted.

3. Innovation:

Strengths

- Tailoring the intervention using self-determination theory, with a focus on autonomy which is developmentally matched. Autonomy supported interventions have been found to be successful in other populations; this study fills a gap related to autonomy-based interventions for young adults with T1D.
- The use of a MOST design is innovative, as it allows for the simultaneous testing of intervention components in an efficient manner. It also is innovative as applied to emerging adults with T1D.

Weaknesses

- As mentioned previously, the individual components (texting, MI) are not novel, but the combination and application represent innovation.

4. Approach:

Strengths

- The scientific rigor of the application is very high.
- Inclusion/exclusion criteria are clear and scientifically appropriate. A strength is to provide minimal exclusion criteria to offer this intervention to as broad an eligible population as possible.
- Specific procedures for enrollment and retention are outlined.
- There is a clear and detailed theoretical framework for the intervention components and resulting outcomes.
- The current trial builds upon and enhances a previously tested intervention with input from the target population.
- The MOST design allows for testing of the individual and synergistic effect of the intervention components on HbA1c.
- The investigators have been highly responsive to the previous critique and have removed the first (untestable) aim, accounted for the role of depression by adding a measure and proposing moderation analyses, revising the analytic plan to maximize power, describing stratified randomization, added measures to assess SDT constructs, clarified the intervention and MOST design decisions.

Weaknesses

- Some concerns were raised by the length of the intervention, and the lack of pilot data specifically among patients with diabetes.
- Some implementation metrics could have been provided such as receipt of text messages, provider interactions.
- Concerns were noted regarding lack of discussion of continuous glucose monitoring and newer technologies.

5. Environment:

Strengths

- The combined resources at Wayne State University (WSU), WSU School of Medicine and the Detroit Medical Center provide adequate clinical and scientific support.
- There is sufficient access to an eligible patient population through two WSU School of Medicine sites: Children’s Hospital of Michigan and in the Division of Endocrinology within the Department of Internal Medicine at the Detroit Medical Center’s University Health Center. Letters of support are provided from referring physicians.

Weaknesses

- None noted.

Study Timeline:

Strengths

- The timeline is detailed and provides adequate information to assess the feasibility of recruiting approximately 100 patients per year (25-30% of the unique patients seen in the clinic). Intervention delivery activities also are provided.

Weaknesses

- None noted.

Protections for Human Subjects:

Acceptable Risks and/or Adequate Protections

- Potential risks and alternative strategies to mitigate risk are provided.

Data and Safety Monitoring Plan (Applicable for Clinical Trials Only):

Acceptable

- A DSMB will be convened. Investigators were responsive to the previous critique in clarifying that a type 1 diabetes expert (physician or equivalent) will be a member of the team; they also have added attrition stoppage rules and recruitment.

Inclusion of Women, Minorities and Children:

- Sex/Gender: Distribution justified scientifically
- Race/Ethnicity: Distribution justified scientifically
- For NIH-Defined Phase III trials, Plans for valid design and analysis: Not applicable
- Inclusion/Exclusion of Children under 18: Including ages <18; justified scientifically
- The study sample will also include emerging adults 18-25. This is justified scientifically

Vertebrate Animals:

Not Applicable (No Vertebrate Animals)

Biohazards:

Acceptable

- Finger stick blood sample collection will be conducted; the study is relying on the clinical collaborator to train staff in the safe collection. More detail on those procedures would have been helpful (minor concern given nature of the data collection).

Resubmission:

- This work was very responsive to previous comments.

Budget and Period of Support:

Budget Modifications Recommended (in amount/time)
Recommended budget modifications or possible overlap identified:

- Reduce conference travel by 25%.
- Computer costs seem excessive. Reduce by 50%.
- No justification is provided for the costs related to the DSMB meeting.
- No justification is provided for the costs associated with intervention content modifications.

CRITIQUE 3

Significance: 1
Investigator(s): 1
Innovation: 1

Approach: 6
Environment: 1

Overall Impact: The proposed project is a resubmission from a new investigator who proposes to use a MOST approach to examine different intervention components designed to enhance the autonomy of older adolescents and emerging adults with T1D. Three theory-based intervention components will be examined including: (1) a question prompt list (QPL) that participants will be directed to with providers during clinic visits, (2) the Motivation Enhancing System (MES) eHealth intervention that is designed to increase motivation for self-management, and (3) diabetes self-care text reminders (TXT). The investigators will examine these different approaches in a full factorial (8-arm) RCT. They will examine the impact of the intervention approaches on HbA1c and diabetes self-management. They will explore the mediating effects of SDT constructs, diabetes clinic visits, and the moderating effects of gender and depression. The result of this research will be an optimized intervention approach that will serve as the foundation for a full-scale effectiveness study. The proposed project addresses a significant public health problem and large gap in the literature regarding transition to autonomous self-management of T1D. A MOST study is an efficient approach to developing an efficacious intervention. The application was highly responsive to the prior review. There are some remaining concerns regarding the intervention components. In particular, the content for the TXT and QPL components have not yet been developed, and their development is not adequately described. It is not clear that the investigators can ascertain exposure to the QPL and TXT components. And since the intervention is quite brief, there are concerns that any combination of the intervention components will be insufficient for engaging participants in better self-management. Closed loop systems are likely to become the standard of care for T1D and, by the time the study concludes, the findings may be out of date. For these reasons, this work offers high moderate impact as written.

1. Significance:

Strengths

- Deterioration of glycemic control in adolescence is well-known and recent research suggests that the problem persists into early adulthood. The investigators argue persuasively that an intervention to support emerging autonomy is a better fit for this population than more traditional approaches reported in the literature.
- The metabolic memory literature shows that periods of poor glycemic control have long-term consequences, even when transient. Consequently, preventing deterioration in adolescents is essential.
- No reports of efficacious interventions to enhance transition to autonomous self-management in this population appear in the literature.
- A MOST approach is the most efficient means to developing an efficacious intervention.

Weaknesses

- None noted.

2. Investigator(s):

Strengths

- The investigative team is excellent. Principal Investigator Carcone is a behavioral researcher with extensive experience with intervention trials youths with T1D and other chronic conditions. She is well-qualified to lead this investigation.
- Co-investigator Ellis is a pediatric psychologist and developer of an MES intervention for youths with T1D.

- Co-investigator MacDonell is a developmental psychologist who has integrated MES with TXT for youths managing asthma.
- Co-investigator Eggly is a communication scientist and has developed a QPL for cancer patients.
- Co-investigator Ghosh is a biostatistician with experience in analysis of data derived from a MOST study.
- Co-investigator Buggs-Saxton in a pediatric endocrinologist.
- The combined group has the content and methods expertise required by the study.

Weaknesses

- None noted.

3. Innovation:

Strengths

- The use of MOST to identify the most useful combination of SDT intervention components is novel.
- Adolescents and emerging adults with T1D is an understudied population.

Weaknesses

- None noted.

4. Approach:

Strengths

- Use of self-determination theory (SDT) is a particularly suitable framework for an intervention designed to enhance autonomy. The investigators have made clear linkages between the intervention components, the SDT constructs (autonomy, competence, and relatedness), and intrinsic motivation to adhere to diabetes self-management requirements and downstream glycemic control.
- The MES has been pilot tested in pre-adolescents, enhances metabolic control, is acceptable, and impacts the hypothesized SDT constructs as expected. A very high (87%) number of preadolescents completed the MES.
- Measurement visits conducted in youths' homes.
- Recruitment, retention, data collection, and procedures to assure data quality and protocol fidelity are very well-developed.
- Well-developed measurement protocol using standardized instruments.

Weaknesses

- The investigators have tested the Question Prompt List only in cancer patients. The QPL has not been used to develop self-management autonomy in youths with T1D. The MES (Motivation Enhancing System) has been tested in T1D participants that are younger than in the proposed study and impact of HbA1c has not been pilot tested. The TXT intervention has been used to increase controller medication in young adults with asthma, not in T1D. Consequently, substantial modifications will be required, particularly in the QPL and TXT components.
- The intervention components, particularly the QPL and TXT, are described in the most general of terms.

- According to Figure 3, the intervention lasts 4-5 weeks. Changing behavior in this patient population is not easy and the investigators provide no preliminary work suggesting that the intervention dose will be sufficient to achieve the desired effects at 3 and 6 months.
- Most recent data indicate that 328 patients will be available for recruitment. It would be useful to know how many new patients are seen each year who meet recruitment criteria as this is a rather low number for a study that proposes to enroll 320.
- There seems to be no way to verify that QPL questions are actually asked of the provider.
- The frequency of missed appointments was described as being high, which raises the concern that the QPL components might not be implemented. Preliminary data on frequency of missed appointments would have been helpful.
- Participants receive standard care visits every 3-4 weeks. If the participant has more than one appointment during the follow-up period, will there be another opportunity to be implement the QPL?
- There seems to be no way to determine if TXT messages were actually read.
- The planned enrollment table indicates 350 participants will be recruited.

5. Environment:

Strengths

- The Wayne State University has the resources required to support the proposed study.

Weaknesses

- None noted.

Study Timeline:

Strengths

- The study timeline is aggressive but may be sufficient to conduct the study as described.

Weaknesses

- None noted.

Protections for Human Subjects:

Unacceptable Risks and/or Inadequate Protections

- Assent procedures for those under 18 years of age are not described

Data and Safety Monitoring Plan (Applicable for Clinical Trials Only):

Acceptable

Inclusion of Women, Minorities and Children:

- Sex/Gender: Distribution justified scientifically
- Race/Ethnicity: Distribution justified scientifically
- For NIH-Defined Phase III trials, Plans for valid design and analysis: Scientifically acceptable
- Inclusion/Exclusion of Children under 18: Including ages <18; justified scientifically

Vertebrate Animals:

Not Applicable (No Vertebrate Animals)

Biohazards:

Not Applicable (No Biohazards)

Resubmission:

- The resubmission was largely responsive to the prior review. However, the description of the intervention remains under-developed.

Budget and Period of Support:

Recommend as Requested

THE FOLLOWING SECTIONS WERE PREPARED BY THE SCIENTIFIC REVIEW OFFICER TO SUMMARIZE THE OUTCOME OF DISCUSSIONS OF THE REVIEW COMMITTEE, OR REVIEWERS' WRITTEN CRITIQUES, ON THE FOLLOWING ISSUES:

PROTECTION OF HUMAN SUBJECTS: UNACCEPTABLE. Reviewers noted that assent procedures for those under 18 years of age are not described.

INCLUSION OF WOMEN PLAN: ACCEPTABLE

INCLUSION OF MINORITIES PLAN: ACCEPTABLE

INCLUSION OF CHILDREN PLAN: ACCEPTABLE

COMMITTEE BUDGET RECOMMENDATIONS: A series of concerns related to the budget included recommendations to 1) Reduce conference travel by 25%, 2) One Principal Investigator requested salary over the NIH cap, 3) Computer costs seem excessive. Reduce by 50%, 4) No justification is provided for the costs related to the DSMB meeting and 5) No justification is provided for the costs associated with intervention content modifications.

Footnotes for 1 R01 DK116901-01A1; PI Name: Carcone, April Marie Idalski

NIH has modified its policy regarding the receipt of resubmissions (amended applications). See Guide Notice NOT-OD-14-074 at http://grants.nih.gov/grants/guide/notice-files/NOT-OD-14-074.html. The impact/priority score is calculated after discussion of an application by averaging the overall scores (1-9) given by all voting reviewers on the committee and multiplying by 10. The criterion scores are submitted prior to the meeting by the individual reviewers assigned to an application, and are not discussed specifically at the review meeting or calculated into the overall impact score. Some applications also receive a percentile ranking. For details on the review process, see http://grants.nih.gov/grants/peer_review_process.htm#scoring.

MEETING ROSTER

Psychosocial Risk and Disease Prevention Study Section
Risk, Prevention and Health Behavior Integrated Review Group
CENTER FOR SCIENTIFIC REVIEW
PRDP
06/11/2018 - 06/12/2018

Notice of NIH Policy to All Applicants: Meeting rosters are provided for information purposes only. Applicant investigators and institutional officials must not communicate directly with study section members about an application before or after the review. Failure to observe this policy will create a serious breach of integrity in the peer review process, and may lead to actions outlined in NOT-OD-14-073 at https://grants.nih.gov/grants/guide/notice-files/NOT-OD-14-073.html and NOT-OD-15-106 at https://grants.nih.gov/grants/guide/notice-files/NOT-OD-15-106.html, including removal of the application from immediate review.

CHAIRPERSON(S)

SPRING, BONNIE, PHD
PROFESSOR
DEPARTMENT OF PREVENTIVE MEDICINE
NORTHWESTERN UNIVERSITY
CHICAGO, IL 60611

MEMBERS

APPELHANS, BRADLEY M, PHD
ASSOCIATE PROFESSOR
DEPARTMENT OF PREVENTIVE MEDICINE
RUSH UNIVERSITY MEDICAL CENTER
CHICAGO, IL 60612

BARKIN, SHARI, MD *
PROFESSOR OF PEDIATRICS
DIVISION OF GENERAL PEDIATRICS
DEPARTMENT OF PEDIATRICS
VANDERBILT UNIVERSITY
NASHVILLE, TN 37232

BEFORT, CHRISTIE, PHD
ASSOCIATE PROFESSOR
DEPARTMENT OF PREVENTIVE MEDICINE
AND PUBLIC HEALTH
UNIVERSITY OF KANSAS MEDICAL CENTER
KANSAS CITY, KS 66160

BOUTELLE, KERRI N, PHD *
PROFESSOR
PEDIATRICS, FAMILY MEDICINE AND PUBLIC HEALTH
UNIVERSITY OF CALIFORNIA, SAN DIEGO
LA JOLLA, CA 92037

BUMAN, MATTHEW P, PHD
ASSOCIATE PROFESSOR
COLLEGE OF HEALTH SOLUTIONS
ARIZONA STATE UNIVERSITY
PHOENIX, AZ 85004

CAAN, BETTE J, DRPH *
SENIOR RESEARCH SCIENTIST
DIVISION OF RESEARCH
KAISER PERMANETE
OAKLAND, CA 94612

DEMARK-WAHNEFRIED, WENDY, PHD
PROFESSOR AND WEBB ENDOWED CHAIR
DEPARTMENT OF NUTRITION SCIENCES
UNIVERSITY OF ALABAMA AT BIRMINGHAM
BIRMINGHAM , AL 35294

DUTTON, GARETH R, PHD
ASSOCIATE PROFESSOR
DEPARTMENT OF MEDICINE
DIVISION OF PREVENTIVE MEDICINE
UNIVERSITY OF ALABAMA AT BIRMINGHAM
BIRMINGHAM, AL 35205

FISCELLA, KEVIN, MD, MPH
PROFESSOR
DEPARTMENT OF FAMILY MEDICINE
SCHOOL OF MEDICINE
UNIVERSITY OF ROCHESTER
ROCHESTER, NY 14620

FITZPATRICK, STEPHANIE LENAY, PHD *
INVESTIGATOR
CENTER FOR HEALTH RESEARCH
KAISER PERMANENTE
PORTLAND, OR 97227

FOCHT, BRIAN CARL, PHD *
PROFESSOR
COLLEGE OF EDUCATION
AND HUMAN ECOLOGY
THE OHIO STATE UNIVERSITY
COLUMBUS, OH 43210

FRANCIS, LORI ANNE, PHD
ASSOCIATE PROFESSOR
DEPARTMENT OF BIOBEHAVIORAL HEALTH
PENNSYLVANIA STATE UNIVERSITY
UNIVERSITY PARK, PA 16802

FUEMMELER, BERNARD F, PHD
PROFESSOR AND GORDON D. GINDER, MD CHAIR IN CANCER RESEARCH
DEPARTMENT OF HEALTH BEHAVIOR AND POLICY
MASSEY CANCER CENTER
VIRGINIA COMMONWEALTH UNIVERSITY
RICHMOND, VA 23298

HOLSEN, LAURA MCGRATH, PHD *
ASSISTANT PROFESSOR
DEPARTMENT OF PSYCHIATRY
HARVARD MEDICAL SCHOOL
HARVARD UNIVERSITY
BOSTON, MA 02115

JAKICIC, JOHN M, PHD
PROFESSOR AND CHAIR
DEPARTMENT OF HEALTH
AND PHYSICAL ACTIVITY
UNIVERSITY OF PITTSBURGH
PITTSBURGH, PA 15261

LEWIS, BETH A, PHD *
PROFESSOR & DIRECTOR
SCHOOL OF KINESIOLOGY
UNIVERSITY OF MINNESOTA
MINNEAPOLIS, MN 55455

LOTH, KATIE ANN, PHD *
ASSISTANT PROFESSOR
DEPARTMENT OF FAMILY MEDICINE
AND COMMUNITY HEALTH
UNIVERSITY OF MINNESOTA
MINNEAPOLIS , MN 55414

MUCCI, LORELEI, SCD *
ASSOCIATE PROFESSOR
DEPARTMENT OF EPIDEMIOLOGY
HARVARD SCHOOL OF PUBLIC HEALTH
HARVARD UNIVERSITY
BOSTON, MA 02115

NAPOLITANO, MELISSA A, PHD
PROFESSOR
DEPARTMENTS OF PREVENTION
AND COMMUNITY HEALTH, EXERCISE
AND NUTRITION SCIENCES
GEORGE WASHINGTON UNIVERSITY
WASHINGTON, DC 20052

NEELON, SARA ELIZABETH, PHD
ASSOCIATE PROFESSOR
DEPARTMENT OF HEALTH
BEHAVIOR AND SOCIETY
JOHNS HOPKINS SCHOOL OF PUBLIC HEALTH
BALTIMORE , MD 21205

NIX, ROBERT, PHD *
ASSOCIATE PROFESSOR
HUMAN DEVELOPMENT AND FAMILY STUDIES
SCHOOL OF HUMAN ECOLOGY
UNIVERSITY OF WISCONSIN-MADISON
MADISON, WI 53706

NOCK, NORA L, PHD
ASSOCIATE PROFESSOR
DEPARTMENT OF EPIDEMIOLOGY
AND BIOSTATISTICS
CASE WESTERN RESERVE UNIVERSITY
CLEVELAND , OH 44106

PAUL, IAN M, MD
PROFESSOR
DEPARTMENT OF PEDIATRICS
COLLEGE OF MEDICINE
PENNSYLVANIA STATE UNIVERSITY
HERSHEY, PA 17033

RAYNOR, HOLLIE A, PHD
PROFESSOR
DEPARTMENT OF NUTRITION
UNIVERSITY OF TENNESSEE
KNOXVILLE, TN 37996

ROSAS, LISA GOLDMAN, PHD *
ASSISTANT PROFESSOR
DIVISIONS OF EPIDEMIOLOGY AND PRIMARY CARE
AND POPULATION HEALTH
RESEARCH INSTITUTE
STANFORD UNIVERSITY SCHOOL OF MEDICINE
PALO ALTO, CA 94305

SAELENS, BRIAN E, PHD *
PROFESSOR
CENTER FOR CHILD HEALTH, BEHAVIOR
AND DEVELOPMENT
SEATTLE CHILDREN'S HOSPITAL RESEARCH INSTITUTE
UNIVERSITY OF WASHINGTON
SEATTLE, WA 98101

SCHNEIDER, MARGARET L, PHD
PROFESSOR
DEPARTMENTS OF URBAN PLANNING AND PUBLIC POLICY
INSTITUTE FOR CLINICAL AND TRANSLATIONAL SCIENCE
UNIVERSITY OF CALIFORNIA, IRVINE
IRVINE, CA 92617

SEVICK, MARY A, SCD
PROFESSOR
DEPARTMENT OF POPULATION HEALTH
SCHOOL OF MEDICINE
NEW YORK UNIVERSITY
NEW YORK, NY 10016

WEISSMAN, RUTH STRIEGEL, PHD *
PROFESSOR
DEPARTMENT OF PSYCHOLOGY
WESLEYAN UNIVERSITY
MIDDLETOWN, CT 06459

WILLIAMS, DAVID M, PHD
ASSOCIATE PROFESSOR
DEPARTMENT OF BEHAVIORAL AND SOCIAL SCIENCES
CENTER FOR HEALTH EQUITY RESEARCH
BROWN UNIVERSITY SCHOOL OF PUBLIC HEALTH
PROVIDENCE, RI 02912

SCIENTIFIC REVIEW OFFICER

FITZSIMMONS, STACEY, PHD, MPH
SCIENTIFIC REVIEW OFFICER
CENTER FOR SCIENTIFIC REVIEW
NATIONAL INSTITUTES OF HEALTH
BETHESDA, MD 20892

EXTRAMURAL SUPPORT ASSISTANT

WALKER, SHEENA
EXTRAMURAL SUPPORT ASSISTANT (INTERN)
CENTER FOR SCIENTIFIC REVIEW
NATIONAL INSTITTUES OF HEALTH
BETHESDA, MD 20892

* Temporary Member. For grant applications, temporary members may participate in the entire meeting or may review only selected applications as needed.

Consultants are required to absent themselves from the room during the review of any application if their presence would constitute or appear to constitute a conflict of interest.
